# The impact of anthelmintic treatment intervention on malaria infection and anaemia in school and preschool children in Magu district, Tanzania: an open label randomised intervention trial

**DOI:** 10.1186/s12879-015-0864-5

**Published:** 2015-03-20

**Authors:** Safari M Kinung’hi, Pascal Magnussen, Coleman Kishamawe, Jim Todd, Birgitte J Vennervald

**Affiliations:** National Institute for Medical Research (NIMR), Mwanza Centre, Isamilo Road, PO Box 1462, Mwanza, Tanzania; Department of Veterinary Disease Biology, Faculty of Health and Medical Sciences, University of Copenhagen, Grønnegårdsvej 15 DK-1870 Frederiksberg C, Copenhagen, Denmark; Depatment of Population Health, London School of Hygiene and Tropical Medicine, Keppel Street, WC1E, 7HT London, UK

**Keywords:** Anthelmintic intervention, Malaria infection, Anaemia, School children, Magu district

## Abstract

**Background:**

Some studies have suggested that helminth infections increase the risk of malaria infection and are associated with increased number of malaria attacks and anaemia. Thus interventions to control helminth infections may have an impact on incidence of clinical malaria and anaemia. The current study assessed the impact of two anthelmintic treatment approaches on malaria infection and on anaemia in school and pre-school children in Magu district, Tanzania.

**Methods:**

A total of 765 children were enrolled into a prospective randomized anthelmintic intervention trial following a baseline study of 1546 children. Enrolled children were randomized to receive either repeated treatment with praziquantel and albendazole four times a year (intervention group, 394 children) or single dose treatment with praziquantel and albendazole once a year (control group, 371 children). Follow up examinations were conducted at 12 and 24 months after baseline to assess the impact of the intervention. Stool and urine samples were collected and examined for schistosome and soil transmitted helminth infections. Blood samples were also collected and examined for malaria parasites and haemoglobin concentrations. Monitoring of clinical malaria attacks was performed at each school during the two years of the intervention.

**Results:**

Out of 1546 children screened for P. falciparum, S. mansoni, S. haematobium, hookworm and T. Trichiura at baseline, 1079 (69.8%) were infected with at least one of the four parasites. There was no significant difference in malaria infection (prevalence, parasite density and frequency of malaria attacks) and in the prevalence of anaemia between the repeated and single dose anthelmintic treatment groups at 12 and 24 months follow up (p > 0.05). However, overall, there was significant improvement in mean haemoglobin concentrations (p < 0.001) from baseline levels of 122.0g/L and 123.0g/L to 136.0g/L and 136.8g/L for the repeated and single dose treatment groups, respectively, at 24 months follow-up which resulted in significant reduction in prevalence of anaemia.

**Conclusions:**

These results suggest that repeated anthelmintic treatment did not have an impact on malaria infection compared to single dose treatment. However, both treatment approaches had overall impact in terms of improvements of haemoglobin levels and hence reductions in prevalence of anaemia.

## Background

Malaria, schistosomiasis and soil transmitted helminth infections (STH) are considered the most important parasitic infections in Sub-Saharan Africa, contributing to a major part of disease burden [1,2]. The diseases share the same geographical distribution and occur as co-infections in humans and thus interact with regards to susceptibility, infection level, and pathology [3-5]. In Tanzania, these infections occur throughout the country and are a major public health problem particularly in school and pre-school age children [5-8]. In school children, the diseases are associated with impaired physical and mental development, impaired learning capabilities, undernutrition and anaemia [2,9,10].

Globally, major control interventions are currently being undertaken to control malaria and helminth infections. These include use of insecticide impregnated bed nets, indoor residual spraying (IRS) and early and effective detection and management of cases using artemisinin based combination therapy for malaria [11-14]. For helminth infections, control interventions include mass drug administration using praziquantel and albendazole, provision of safe water, improved sanitation and health education [2,15].

Some previous studies have demonstrated that helminth infections may increase the risk of infections with *P. falciparum* [16-20] and other infections such as HIV and TB [21,22]. However other studies have not found this relationship [23]. Another important aspect of malaria and helminth co-infections in humans is their joint contribution to anaemia [24]. A study in Nepal [24] observed that *P. vivax* malaria and hookworm co-infections in pregnant women were associated with more frequent malaria attacks and severe anaemia than seen in women who harbour only one parasite infection. In the Philippines, it was demonstrated that even at low infection intensities, multiple parasite infections enhance the risk of anaemia [25,26]. Thus interventions to control helminth infections in areas where helminth infections are co-endemic with malaria could be expected to have not only an impact on prevalence and intensity of helminth infections but also an impact on disease burden due to malaria [27]. However there have been few longitudinal randomized studies to test these hypotheses [28-30]. Most studies have been cross sectional in nature and hence more longitudinal randomized studies are needed.

The objective of this study was therefore to assess the impact of an anthelmintic intervention delivered through two different approaches on malaria infection and on anaemia. The study was designed to test the hypothesis that helminth infections modulate immune responses against *P. falciparum* infection in an infection intensity dependent manner and thus increase susceptibility to malaria infection and related morbidity. Thus control of helminth infections would enhance immune response against *P. falciparum* malaria and reduce incidence of clinical malaria attacks and anaemia.

## Methods

### Study area and population

The study was implemented in Magu district, Mwanza region, North-western Tanzania, and involved six primary schools namely Mwamayombo, Nyashimo, Bulima, Milambi, Ihale and Ijitu. From each school, school children aged 6–13 years (grades I-V) were randomly selected and included in the study. The aim was to get at least 100 children per school. Where the required number of 100 school children was not reached, pre-school children aged 3–5 years were also enrolled into the study. Selection of pre-school children was made conveniently whereby parents and guardians living in the community surrounding each school were requested to bring their children to the examination centre.

### Study design, randomization and treatments

The study was a longitudinal open label intervention trial which was preceded by cross-sectional baseline study that enrolled 1546 children in the catchment areas of the 6 primary schools. During the baseline study, the prevalence and infection intensity of *P. falciparum, S. mansoni, S. haematobium,* hookworm and *T. trichiura* was determined using standard methods as described in Kinung’hi *et al.* [5]. Haemoglobin concentrations were also assessed using the Haemocue method. Quality control was performed by re-examining 10% randomly selected blood slides, urine filters and Kato smears by an experienced independent technician.

Following the baseline study, 765 children were selected and included in the longitudinal study. The inclusion criteria were: Being pre-school children (age 3 to 5 years) or school children (age 6 to 13 years) living in the selected villages for at least one year. In addition, selected children were those infected with either *S. mansoni or S. haematobium* or both. Exclusion criteria were: Children who had stayed in the village for less than one year, children whose parents or legal guardians did not provide informed consent and children with severe malaria and anaemia. Severe malaria was defined as a seriously sick child presenting with fever (axillary temperature ≥ 37.5) plus one or more signs of seizures, respiratory distress, prostration, impaired consciousness or coma combined with the presence of *P. falciparum* parasitemia but in the absence of other causes of febrile illness [31,32]. Severe anaemia was defined as a child with haemoglobin concentrations below 80g/L. Children who had received any anthelminthic treatment within one year before baseline were also excluded from the study.

Haemoglobin concentrations were taken as the reference variable for calculation of sample size. A minimum sample size of 620 children (310 children for the repeated anthelmintic treatment group and 310 children for the single dose treatment group) was considered sufficient to give a power of 90% to be able to detect a significant difference of at least 5g/L between the two groups at 95% confidence interval and 5% level of precision. However, after baseline survey and assessment for inclusion criteria, 765 children were selected and randomized into either the repeated anthelmintic treatment group (intervention group, 394 children) or the single dose anthelmintic treatment group (control group, 371 children). Generation of random allocation sequence and assignment of participating children to intervention and control groups was performed by an experienced statistician.

Children in the intervention group were treated with repeated doses of praziquantel 40mg/kg and albendazole 400mg four times a year at three months interval while children in the control group were treated with a single dose of praziquantel 40mg/kg and albendazole 400mg once a year. The two treatment approaches for the intervention and control groups were based on the assumption that the intensive repeated treatment (four times a year) of the intervention group will make this group helminth free while for the control group, the standard single dose treatment (once per year) will allow for re-infection to take place in 50% or more of children in this group [33-35]. Both praziquantel and albendazole were supplied by the medical stores department of the ministry of health and social welfare (MoHSW), Tanzania. Treatments took place at the school premises and were administered by a qualified medical doctor assisted by a nurse and trained school teachers. Before taking the drugs, children were given a piece of bread and a soft drink (fruit juices) to minimise side effects of praziquantel. While albendazole was given as a single dose at a dosage rate of 400mg, praziquantel was administered according to weight. All drugs were taken orally together with clean safe drinking water under direct observation. After taking the drugs, children were allowed to rest for a period of 30–60 minutes during which side effects related to praziquantel treatment were monitored. Overall praziquantel was well tolerated except for mild abdominal discomforts that lasted for about 30 minutes.

**Figure 1:**
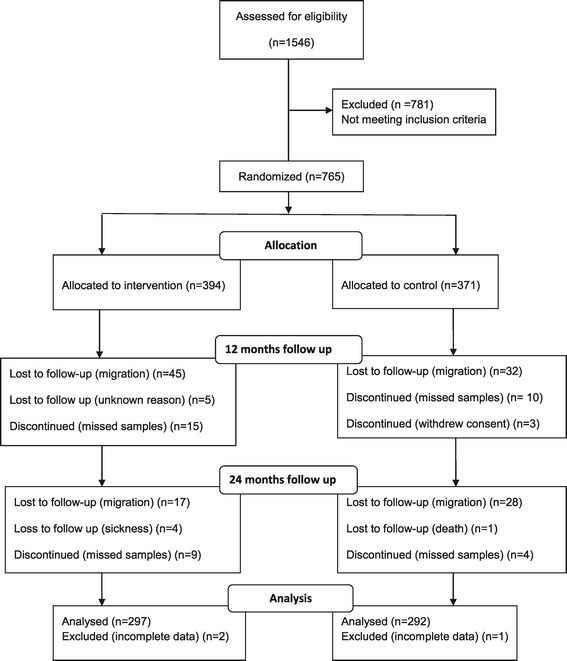
**Study flow diagram.**

The cohort of 765 children was followed up at 12 and 24 months after baseline. During these two follow up time points, the prevalence and infection intensity of *P. falciparum, S. mansoni, S. haematobium* and hookworm as well as haemoglobin concentrations were assessed. The 12 months follow up assessment took place between October and November 2007 and included 655 children. The 24 months follow up assessment took place between October and November 2008 and involved 592 children. Of the 592 children who completed both the 12 months and the 24 months surveys, 297 were from the intervention group while 292 were from the control group. This amounted to a compliance rate of 75.4% and 78.8%, respectively. This study is registered on ClinicalTrials.gov, registration number NCT00347113; registered June 30, 2006 and reported according to CONSORT guidelines for randomized trials [36,37]. The study flow chart is shown in Figure 1.

Major reasons for loss to follow-up included migration from the study area, missing a second stool or urine sample, withdrawal of consent, sickness during follow-up survey days and death.

### Monitoring of clinical malaria attacks

During the 24 months follow-up period, the incidence of clinical malaria attacks was monitored at each primary school assisted by trained school teachers. Monitoring of incidence of clinical malaria attacks was done for all children included in the longitudinal study by passive case detection whereby children were asked to report to teachers when they had febrile illness. The parents and guardians of all children enrolled in this study were also informed about the study and requested to report to teachers when their children had febrile illness. The teachers were trained on how to make presumptive diagnosis and treatment of malaria cases. They were also trained to collect finger prick blood, prepare and stain thick blood smears using the Giemsa stain method and keep records. A register of malaria cases was established at each primary school. A laboratory technician visited each school on a weekly basis to collect the Giemsa stained blood smears and all information recorded. Blood smear examination was undertaken at the parasitology laboratory of the National Institute for Medical Research (NIMR), Mwanza Centre. School children found with febrile illness were treated with Artemether-Lumefantrine which was the first line antimalarial drug at the time of the study or were referred to nearby health facilities according to national guidelines on malaria treatment. A malaria episode (case) was defined as a child presenting with febrile illness with axillary body temperature ≥37.5°C plus a positive thick blood smear for malaria parasites in the absence of signs for other infectious diseases.

### Study outcomes

The following study outcome measures for malaria infection and anaemia were assessed during the study:

- Prevalence of malaria parasitaemia defined as the proportion of children with a positive blood smear for malaria parasites by the Giemsa stain method.
- Malaria parasite density defined as the number of malaria parasites per microlitre (μL) of blood calculated assuming 8000 white blood cells/μL of blood and estimated by counting the number of parasites per 200 leucocytes in Giemsa stained thick films at 100× magnification. Malaria parasite density was classified as low (<5000 parasites/μL of blood) and high (≥5000 parasites/μL of blood).
- Incidence of clinical malaria attacks defined as number of new cases of children presenting with febrile illness with axillary body temperature ≥37.5°C plus a positive thick blood smear for malaria parasites by Giemsa stain method in the absence of signs for other infectious diseases. The incidence of clinical malaria attacks was observed for the entire duration of the intervention (two years).
- Prevalence of anaemia and severe anaemia defined as the proportion of children with haemoglobin (Hb) concentrations less that 120 g/L and 80 g/L, respectively, measured by the Haemocue method.

### Ethical statement

The study was approved by the Medical Research Coordination Committee (MRCC) of the National Institute for Medical Research (NIMR), Tanzania (Reference No. NIMR/HQ/R.8a/Vol. IX/355). Before commencement of the study, the research team conducted meetings with village leaders, teachers and community members of all selected villages. During these meetings, the objectives and procedures of the study were explained including study benefits and potential risks and discomforts. Written informed consent for children who participated in the study was sought from parents or legal guardians after they had been informed about the study. Children were also requested to give written assent and were informed of their right to refuse to participate in the study and to withdraw at any time during the study without jeopardizing their right of access to other health services. Invasive procedures such as collection of blood samples were explained to parents and children and were carried out using sterile disposable materials. All children found infected with any of the parasites *S. mansoni, S. haematobium,* soil-transmitted helminthiasis and *P. falciparum* and those found with ailments not targeted by the project were treated free of charge according to national guidelines. Study identification numbers were used instead of children names and information collected was kept confidential. Feedback to the study population in the form of dissemination workshops was conducted during the course of the study.

### Data management and statistical analysis

Data were double entered into Dbase V software (Borland International, Scotts Valley, California, USA) and analyzed using STATA Version 10 (STATA Corp., Texas, USA). Infection intensities (of positive samples only) were calculated as geometric mean of parasites per microlitre of blood for *P. falciparum* and geometric mean of eggs per gram of faeces for *S. mansoni* and hookworms. For *S. haematobium*, infection intensity was calculated as geometric mean of eggs per 10ml of urine. For each survey point (baseline, 12 months follow up and 24 months follow up), the Chi-square test was used to compare proportions (prevalence) of malaria infection and prevalence of anaemia between the intervention and control groups. Likewise, for each survey point, the student’s t-test was used to compare geometric mean parasite counts and mean haemoglobin concentrations between the intervention and control groups where two groups were compared. The repeated measures analysis of variance (rmANOVA) was used to compare geometric mean parasite counts and mean haemoglobin concentrations where repeated measurements for all three survey points (baseline, 12 months follow up and 24 months follow up) were compared longitudinally. Comparison of the incidence (or risk) of clinical malaria attack among children in the intervention group and those in the control group was performed by calculating the relative risk (risk ratio) using 2×2 contingency tables. Tests were considered statistically significant at p < 0.05.

## Results

### Baseline characteristics

Table 1 shows baseline characteristics of children included in the longitudinal study. There were no significant differences in baseline characteristics of children in the two groups.

**Table 1:** **Baseline characteristics of children included in the trial by randomization group (n = 765)**

**Variable**	**Intervention group (n = 394)**	**Control group (n = 371)**
**Sex**		
Boys (n = 392)	193 (49.0%)	180 (48.5%)
Girls (n = 373)	201 (51.0%)	191 (49.0%)
**Age distribution (years)**		
3 – 5 (n = 93)	40 (10.1%)	53 (14.3%)
6 – 8 (n = 517)	278 (70.6%)	239 (64.4%)
9 – 13 (n = 155)	76 (19.3%)	79 (21.3%)
**School**		
Mwamayombo	52 (51.5)	49 (48.5)
Nyashimo	85 (50.6)	83 (49.4)
Bulima	68 (50.7)	66 (49.3)
Milambi	56 (53.8)	48 (46.2)
Ihale	75 (51.4)	71 (48.6)
Ijitu	58 (51.8)	54 (48.2)
All schools	394 (51.5)	371(48.5)
***S. mansoni*** **infection**		
Prevalence (%)	238 (60.4)	220 (59.3)
Intensity (Epg)*	49 (42–59)	50 (42–59)
***S. haematobium*** **infection**		
Prevalence (%)	90 (22.8)	85 (22.9)
Intensity (Eggs/10 ml)*	15 (11–20)	17 (13–23)
***S. mansoni/S. haematobium co-infection***		
Prevalence (%)	65 (16.6)	61 (16.4)
*S. mansoni* intensity (Epg)*	31 (23–42)	41 (29–58)
*S. haematobium* intensity (egg/10 ml)*	19 (11–28)	16 (10–26)
**Malaria infection**		
Prevalence (%)	123 (31.2)	123 (33.2)
Parasite density (mps/μL)*	530 (414–676)	510 (403–644)
**Hookworm infection**		
Prevalence (%)	80 (20.3)	68 (18.3)
Intensity (Epg)*	54 (39–76)	53 (39–73)
Prevalence of anaemia (%)	142 (36.7)	134 (35.5)
Prevalece of severe anaemia (%)	5 (1.3)	4 (1.1)
Mean haemoglobin level (g/L)**	122 (121–123)	123 (122–124)
**Multiple infections (%)**		
Single parasite	199 (50.5)	186 (50.1)
Two parasites	148 (37.6)	136 (36.7)
Thee/four parasites	47 (12.0)	49 (13.2)

*Infection intensity expressed as geometric mean parasite counts of positive samples only with 95% confidence interval shown in brackets.

**Mean haemoglobin concentrations with 95% confidence interval shown in brackets.

Out of the 765 children included in the longitudinal study, 589 (77.0%) completed the two year follow up period and were included in the analysis of whom 291 (49.4%) were boys. Mean age was 9 years. Out of the 589 children who completed the 24 months follow up, 302 (51.4%) were infected with at least one of the parasites *P. falciparum, S. mansoni, S. haematobium,* Hookworm and *T. trichiura*. Children who were infected with *P. falciparum* were 144 (24.5%), out of whom 55 (38.2%) had helminth co-infections. Only 3 children (0.5%) had *T. Trichiura* infection and hence *T. Trichiura* infection was excluded from further analysis.

### Effect of the intervention on malaria infection, haemoglobin concentrations and anaemia

There was no significant difference in malaria infection (prevalence and malaria parasite density) and in the prevalence of anaemia between the intervention and control groups at 12 and 24 months follow up period (p > 0.05) (Table 2). However, overall, there was a significant improvement in mean haemoglobin concentrations (F = 96.47, p < 0.001) from baseline levels of 122.0g/L (95% CI 120.5-123.4) and 123.0g/L (95% CI 121.6-124.5), for the intervention and control groups, respectively, to 136.0g/L (95% CI 134.2-137.8) and 136.8g/L (95% CI 135.0-137.7) for the intervention and control groups, respectively, at the 24 months follow-up period which resulted in a significant reduction in the prevalence of anaemia over the two years period but without significant differences between intervention and control groups (Table 2).

**Table 2:** **Comparison of overall prevalence and geometric mean parasite density of malaria infection, mean Hb levels and anaemia after one and two years of anthelmintic treatment by randomization groups**

**Variable/randomization group**	**Baseline**	**12 months follow up**	**24 months follow up**
Prevalence of *P. falciparum* infection (n,%)			
Intervention	123 (33.2)	145 (44.9)	74 (24.9)
Control	123 (31.2)	137 (42.4)	70 (24.0)
*p-value*	*0.567*	*0.526*	*0.790*
Intensity of *P. falciparum* infection (95% CI)			
Intervention	530 (414-678)	833 (664-1048)	326 (279-382)
Control	510 (403-645)	906 (724-1133)	343 (294-400)
*p-value*	*0.825*	*0.608*	*0.658*
Prevalence of anaemia (n,%)			
Intervention	142 (36.0)	120 (37.2)	43 (14.5)
Control	134 (36.1)	112 (34.7)	38 (13.0)
*p-value*	*0.971*	*0.806*	*0.634*
Prevalence of severe anaemia (n,%)			
Intervention	5 (1.3)	1 (0.3)	-
Control	4 (1.1)	1 (0.3)	-
*p-value*	*NA*	*NA*	*NA*
Mean haemoglobin level in g/L (95% CI)			
Intervention	122.0 (120.5-123.4)	123.5 (121.9-125.0)	136.0 (134.2-137.8)
Control	123.0 (121.6-124.5)	123.2 (121.7-124.6)	136.8 (135.0-137.7)
*p-value*	*0.270*	*0.749*	*0.494*

Baseline prevalence of heavy *P. falciparum* infection (≥5000 mps/μL) was 2.1% and 2.2% for the intervention and control group, respectively and was reduced to 0% at the end of the intervention. This represented a significant reduction over the two years follow up period but without significant differences between groups (χ^2^ = 0.07, p = 0.790). Likewise, there was an overall significant reduction in malaria parasite density (F = 32.94, p < 0.001) from baseline levels of 530 (95% CI 414–678) and 510 (95% CI 403–645), for the intervention and control groups, respectively, to 326 (95% CI 279–382) and 343 (95% CI 294–400) for the intervention and control groups, respectively, at the 24 months follow-up period but without significant differences between groups (t = 0.44, p = 0.658) (Table 2). However, for all groups, there was an increase in malaria prevalence and malaria parasite density from baseline to 12 months follow up (Table 2) which could be a result of other factors such as climate variability and seasonality of malaria transmission.

### Incidence of clinical malaria cases

Out of the 765 children originally included in the longitudinal follow up, 589 (77.0%) children completed the two year follow up period for malaria attacks (297 were in the intervention group while 292 were in the control group). A total of 221 children presented with febrile illness suggestive of malaria of whom 114 were from the intervention group and 107 were from the control group. However only 85 children (38.5%) met the criteria of confirmed malaria cases (axillary temp ≥37.5% plus a blood smear positive for malaria parasites). Forty confirmed malaria cases were reported from the intervention group while 45 confirmed malaria cases were reported from the control group. Eighteen children (21.2%) reported more than one episode of clinical malaria attacks (2–4) per year during the first year of the intervention. However, this occurred less frequently during the second year of the intervention whereby only 7 children (8.2%) reported more than one episode of clinical malaria attacks with the highest frequency of 2 attacks per child per year. There was clustering of overall clinical malaria attacks by school which corresponded with baseline malaria prevalence at respective schools (data not shown).

Overall, there was no significant differences in the incidence of clinical malaria attacks (RR = 1.144, 95% CI 0.759-1.725, p = 0.519) or malaria parasite density (t = −0.76, p = 451) between children in the intervention group and those in the control group (Table 3).

**Table 3:** **Malaria case incidence and malaria parasite density by treatment group over 24 months follow up period**

**Variable**	**Intervention group (n = 297)**	**Control group (n = 292)**	**P-value**
Malaria case incidence*	6.7 (4.8-8.9)	7.7 (5.7-10.1)	0.519
Malaria parasite density**	2658 (1647-4291)	3100 (1831-5249)	0.451

*Malaria case incidence defined as the number of new clinical malaria attacks observed in a given treatment group per 100 person years with 95% confidence intervals shown in brackets.

**Malaria parasite density expressed as geometric mean malaria parasites per microlitre of blood (mps/μL) (positive samples only) with 95% confidence intervals shown in brackets.

## Discussion

Results of the current study suggest that repeated anthelmintic treatment (treatment with praziquantel and albendazole four times a year) did not have any impact on malaria infection (prevalence, malaria parasite density and frequency of malaria attacks) compared to the single dose annual treatment as no differences were observed between children in the repeated anthelmintic treatment (intervention) group compared to children in single dose annual treatment (control) group. This is contrary to what was expected. Previous studies had observed that helminth infections increases the risk of malaria infection [17-19,38,39] which could imply that an anthelmintic treatment intervention would result in reduced frequency of malaria attacks and/or malaria parasite densities in areas where both malaria and helminth infections are co-endemic [40]. One explanation of this finding could be that the impact of helminth infections on malaria infection is intensity dependent such that heavy helminth infections are required to induce a shift in Th1/Th2 balance which in turn would be reflected in differences in the observed malaria infection between children in the intervention and control groups. This view is supported by findings of Sokhna et al. [18], Fenton [41] and Wiria et al. [42]. Sokhna *et al.* [18] observed that the incidence rate of malaria attacks was significantly higher in Senegalese children infected with *S. mansoni* particularly those carrying the highest egg loads as compared to uninfected children. Fenton [41] observed that the outcomes of coinfection are highly dependent on helminth burden among other factors. Another recent study by Waknine-Grinber *et al.* [43] demonstrated that concomitant *S. mansoni* infection reduced the incidence of cerebral malaria in *P. bergheii* infected mice but the effect was dependent on *S. mansoni* parasite load. As most of the helminth infections in this study were light, their impact on malaraia infections might have been weak. The results are however in agreement with findings of Beasley *et al.* [44] who worked with school children aged 7–12 years in Tanga Region in Northeast Tanzania where malaria, *S. haematobium* and STHs are co-endemic. He observed that 15 to 16 weeks following anthelmintic treatment using praziquantel and albendazole, children in the treatment group had significant reductions in prevalence and infection intensity of *S. haematobium* and STHs and in the prevalence of anaemia compared to children in the control (placebo) group but did not differ in terms of prevalence and infection intensity of *P. falciparum* malaria. Another important observation was the relatively few confirmed malaria attacks relative to the number of cases who reported with febrile illness. Out of the 221 reported cases of febrile illness, only 85 (38.5%) were confirmed to be due to *P. falciparum* infection. This observation suggests that there are other causes of febrile illness in this study area. Similar observations have been made in other parts of Tanzania [45-47] and hence there is a need for further longitudinal studies.

As observed by previous studies investigating the association between malaria and helminth infections [23,48], one weakness of the design of the current study is the lack of a proper untreated control group due to ethical reasons. While children in the intervention group were treated with praziquantel 40mg/kg and albendazole 400mg four times a year, children in the comparison group had to be treated using the minimum recommended single dose of praziquantel 40mg/kg and albendazole 400mg annually according to the national schistosomiasis and soil-transmitted helminthiasis treatment guidelines in Tanzania. Although it was assumed that the single dose annual anthelminthic treatment of children in the control group would allow for re-infection of 50% or more of children in this group and that this level of helminth re-infection would still induce the same effect on malaria infection, the treatment might have contributed to lack of differences in malaria infection between children in the intervention group compared to those in the control group. Despite the lack of significant differences between the study groups, there was an overall reduction in prevalence of malaria parasitaemia, intensity of infection and number of malaria attacks from baseline through to the 12 months and 24 months follow up surveys. However, this reduction was not consistent over time since the prevalence and infection intensity of malaria infection was higher at the first (12 months) follow up survey. The fact that this reduction was not consistent over time suggests that other factors such as seasonal variation in malaria transmission resulting from malaria vector dynamics and climatic/environmental factors might have acted as confounding factors and contributed to the overall reduction observed.

Earlier studies to investigate the association between malaria and helminth infections using a randomized design include the study of Murray *et al.* [49] which suggested that treatment of children with helminth infections (*A. lumbricoides*) resulted into an increase of malaria cases. Two further studies with similar design were conducted in Madagascar [50,51] and showed that children aged more than 5 years treated for helminths (*A. lumbricoides*) had a significant increase in malaria parasite densities compared to untreated controls suggesting a negative interaction between *A. lumbricoides* infection and malaria parasite density. It might be difficult to compare findings of the current study with the studies of Murray and Brutus. While the study of Murray *et al.* [49] included a very small sample size (only 35 children were studied), all the three studies [49-51] investigated the association between *P. falciparum* and *A. lumbricoides* infections which involved a different helminth specie (*A. lumbricoides*) from the helminth species investigated in the current study (*S. mansoni*, *S. haematobium* and hookworms). Further, the studies of Murray and Brutus were conducted in a different epidemiological setting (and hence different disease transmission patterns) and used different treatment regimen against helminth infections. While Murray *et al.* [49] used piperazine to treat STH infections, Brutus *et al.* [50,51] used levamisole to treat STH infections. No treatment was given against schistosome infections.

Likewise, there were no significant differences in prevalence of anaemia or in haemoglobin levels between children in the intervention group compared to children in the control group over the two years assessment periods. However, for both groups, there was significant improvement in mean haemoglobin levels and hence a reduction in prevalence of anaemia. The observed changes in haemoglobin levels and the prevalence of anaemia followed a similar trend as changes in the prevalence and infection intensity of malaria suggesting that malaria infection was the most important contributor to anaemia in the studied population in line with findings of other studies [52,53]. Further, the anthelmintic intervention might have contributed to the improvement in haemoglobin levels and hence reduction in the prevalence of anaemia by acting through reductions in prevalence and infection intensity of helminth infections (*S. mansoni*, *S. haematobium* and hookworms) and the prevalence and infection intensity of *P. falciparum* infection in line with reports by other investigators [27,44,54,55]. This argument is supported by baseline findings [5] whereby multivariate analysis showed that *P. falciparum* and *S. haematobium* infections were the most important predictors of anaemia in the studied population. Other studies which have provided evidence on the importance of malaria infections on anaemia include studies on interventions directed against malaria such as Insecticide Treated Nets (ITNs) and chemoprophylaxis. Results of these studies demonstrated increases in haemoglobin levels and a strong impact on anaemia [56,57]. The most striking impact of the anthelmintic intervention was on severe anaemia which was reduced by 100% for both the intervention and control groups.

## Conclusions

In conclusion, findings of the current study show that repeated anthelmintic treatment did not have a direct impact on malaria infection (prevalence, malaria parasite density and frequency of malaria attacks) as compared to single dose annual treatment. However, both treatments had an overall impact in terms of improvements of haemoglobin levels and hence reductions in prevalence of anaemia.
